# Reporting of blood pressure levels and self-monitoring practices: a survey among outpatients diagnosed with hypertension in Bogotá, Colombia

**DOI:** 10.1186/s12875-023-02111-8

**Published:** 2023-09-14

**Authors:** Juan Carlos Villar, Skarlet Marcell Vásquez, Angela Manuela Balcázar, Luz Angela Torres López, Edgar Camilo Barrera, Angélica María Moreno

**Affiliations:** 1 Centro de Investigaciones, Fundación Cardioinfantil – Instituto de Cardiología, Calle 163 A # 13B – 60 Torre H, Piso 3, Bogotá, Colombia; 2 Facultad de Ciencias de La Salud, Universidad Autónoma de Bucaramanga, Avenida 42 #48 - 11, Bucaramanga, Santander Colombia

**Keywords:** Hypertension, Blood pressure, Self-care, Blood pressure monitoring, Home blood pressure monitoring, Health literacy, Primary care, Cross-sectional study, Self-report

## Abstract

**Background:**

Routine blood pressure (BP) self-monitoring is recommended for patients already diagnosed with hypertension. How often these patients can report their BP levels is unknown, particularly in low-and-middle income countries.

**Methods:**

We surveyed (January 2021 to May 2022) representative samples of patients with established diagnosis of hypertension from 3 health care networks (involving 74 outpatient clinics) and 2 university hospitals in Bogotá, Colombia. Trained health care professionals conducted a telephone survey including questions on demographics, medical history, and general understanding about hypertension and its potential complications. The outcome variables were the self-report of participant’s BP levels (primary) and monitoring practices among participants.

**Results:**

Out of 2609 consecutively contacted patients sampled from institutional records, 2323 were invited and 1566 (mean age 66.5, SD = 12.1 years, 74.4% females, 64.0% living low socio-economic strata) gave consent to participate. While 66% of participants had over 5 years of diagnosis, 39.5% had most (≥ 60%) of their follow-up visits with the same doctor. Overall, 645 (41.5%, 95%CI 39.1 -43.9) participants reported their BP levels. This proportion was independent of time from diagnosis, but higher among those of younger age, living in higher socio-economic strata, having more years of education and using more information technologies. Also, more patients reported their BP levels if seen ≥ 60% of the times by the same physician (43.4% Vs. 36.7%). Those reporting closer BP self-monitoring more often used electronic devices, received 2 + medications, and had better knowledge about hypertension.

**Conclusion:**

A minority of hypertensive patients seen in Bogotá were aware of their own BP levels. Those in such capacity were in a better social position, more often seen by the same doctor, knew their condition better and handled more complex treatments. Hypertensive patients from Bogotá may benefit from a more continuous medical care, patient education programs and promoting BP home monitoring.

**Supplementary Information:**

The online version contains supplementary material available at 10.1186/s12875-023-02111-8.

## Introduction

Globally, hypertension is the leading risk factor in terms of burden of disease, because of its relationship with major vascular events and other complications [1]. The strength of this association parallels with blood pressure (BP) levels and decreases with treatment intensity [2]. Despite this linear relationship, many medical associations and guidelines establish BP thresholds to facilitate risk stratification and handle treatment goals for day-to-day patient care [3]. Still, many diagnosed patients do not reach such goals, even though effective, affordable treatment is widely available.

Several studies, both in high and low-and middle-income countries (LMIC), have reported the rates of awareness, treatment (among those aware) and control (achieving goals among those treated) in unselected populations [4–11]. The results, disappointing in general, are even more for LMIC, including Colombia, where social and ethnic disparities widen such health care gaps [12, 13].

Fewer studies have explored, among patients already diagnosed, their knowledge of BP levels [14, 15]. As most hypertensive patients will need long-term medication, often drug combinations, self-monitoring of BP levels is recommended to assist physicians in adjusting their treatment. Scientific societies have issued guidelines serving that purpose through appropriate measurements [16, 17]. Self-reported BP levels seem reliable and useful [18], especially if taken from BP electronic monitoring devices. Also, BP home monitoring is associated with both treatment adherence and meeting goals [19].

In Colombia, hypertension with a prevalence of 23% of adults, was the leading cause of outpatient visits recorded in the most recent (2007) national health survey [20]. A reform to the country’s health care system introduced in 1993 increased medical coverage and reduced out-of-pocket expenses [21]. Under the current system, Colombian citizens are insured through two funding mechanisms: either by making direct, income-adjusted payments to the system (the so-called contributory regime), or through government funds transferred to the system to cover those with lower or no regular income (the so-called subsidized regime). Public health care facilities provide care mostly to patients in the subsidized regime. Despite reaching an almost universal health care coverage (99.1% of the 51.8 million Colombians covered in 2022 [22]), getting continuous, articulated medical attention is still a day-to-day challenge for many, especially low-income or rural populations.

National estimates showed further increased in prevalence in the previous decade [23]. Furthermore, recent community-based studies reported that half of hypertensive adults are still unaware of their condition, and only 37% of those diagnosed were meeting treatment goals [4, 24]. Both the high burden of hypertension and the large potential for improvement in outpatient care prompt us to understand the context of this condition, particularly from the patient's perspective.

There are few data on how often patients already diagnosed know their BP levels in Colombia. That figure may speak of understanding hypertension, the frequency and adequacy of BP self-monitoring, and other key variables around the care for hypertensive patients. We therefore surveyed patients with diagnosis of hypertension from diverse health care facilities in Bogotá (8 million inhabitants), the capital and largest city in Colombia. We sought to estimate the proportion of patients with knowledge of BP levels, and to explore its variations across factors such as the time from being diagnosed, the continuity of their medical follow up, patterns of self-monitoring and general understanding of their condition.

## Methods

This cross-sectional study is part of the “*Vector Salud Bogotá”* project [25]. It comprised a baseline assessment of the hypertensive patients who will be part of a cluster randomised trial, intended to evaluate changes in knowledge, attitudes and practices after dissemination of evidence-based recommendations. The larger project sought to assemble clusters of outpatients seen at the same site, whose main diagnosis was one of seven conditions (e.g., hypertension, diabetes, heart failure, etc.). The overall goal was to have 250 clusters of around 20 patients each, at least 70% from public health care facilities and 30% with hypertension as target condition. That is, the expected hypertension subpopulation for this study was around 1500 patients, distributed in approximately 75 clusters of patients from a similar number of sites.

The project invited public and private health care networks and university hospitals in Bogotá, seeking to facilitate the enrollment of adult patients with established diagnosis of hypertension, who live in the city. Upon approvals from administrators and Institutional Review Boards (IRBs), participating institutions sent to the project lists of potentially eligible patients from the previous year. Research assistants consecutively contacted by phone patients from each site using the provided lists until clusters (with the intended goal one for each site) were assembled. During the call, the assistants explained the purpose of the project, confirmed their eligibility and explored their interest to take part in the project, updating and expanding their contact information.

In a second call, the candidates confirmed their consent and if so, research assistants applied the survey. It included questions on patterns of care (location, frequency of follow-up visits) hypertension treatment and knowledge of their own BP levels. Study personnel asked participants reporting that knowledge to recall their actual BP levels when last checked out of the medical office, how long ago it was, and the usual method of monitoring (either using electronic devices, or the auscultatory method). In a third call, participants answered questions on general understanding of hypertension. This section was also developed by the project team, following reviews for face validity, based on previous local surveys and the national guidelines for Hypertension. It included 5 questions on the meaning of the term hypertension and of the two numbers expressing the BP, treatment goals; the need to adjust treatments when out of target, and the organs potentially affected by hypertension (see Supplementary file). Our questionnaire also explored patients’ knowledge on specific recommendations, but they are not part of this report.

The consecutive contact and sequence of calls continued until the project enrolled 20–25 patients for each health care facility, whether an outpatient clinic of a health care network, or a university hospital.

Our primary outcome variable was the participant report of his/her own BP levels. Our secondary outcomes were a) the report of BP self-monitoring, including time since last check and method of measurement and b) general understanding of hypertension (4 questions, as described above). Independent variables of interest were socio-demographics, and patterns of care, particularly frequency of follow-up visits done with the same doctor and intensity of treatment using the reported number of prescribed medications.

This report included mostly descriptive statistics. Continuous variables are reported as means with their standard deviation (except for time variables, described as means and interquartile range), and categorical values as counts and percentages. For hypothesis testing (e.g., when describing the characteristics of participants with/without knowledge of their BP levels), we used student’s T tests for comparing means from independent groups, or Chi Square statistics for comparing frequencies across categories.

## Results

**Fig. 1:**
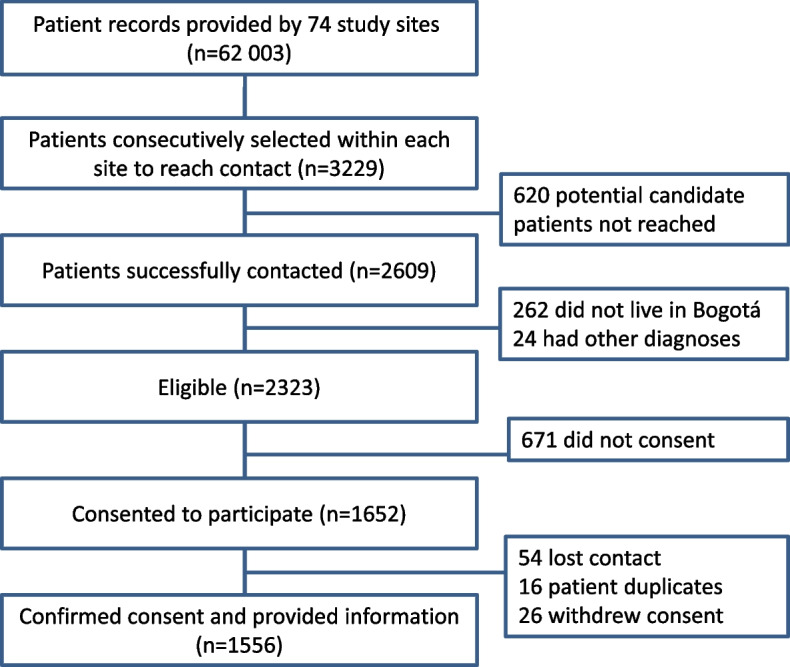
Flowchart of the enrollment process

The collaborating institutions for this study provided lists with 62 003 potential candidates from 74 outpatient clinics (56 from two public health care networks; 16 from one private health care network and 2 from university hospitals, one public and one private). Out of 2609 patients consecutively contacted at each site, 1556 consenting participants (21 per site on average, 75.6% from public health care networks, 21.6% from the private health care network, and 2.8% from university hospitals) were surveyed from January 18, 2021, to May 25, 2022. The flowchart in Fig. 1 shows the enrollment process.

Table 1 describes general characteristics of participants. The mean age was 66.5 years, and they were mostly (74.4%) female patients with a mean body mass index of 27 kg/m^2^. The majority (64%) lived in low socio-economic strata neighborhoods and had up to 5 years of formal education (63.1%). Among the co-existing health conditions reported, dyslipidemia (28.3%) and diabetes mellitus (18.3%) were the most common. Although most (96.6%) participants reported access to a cell phone, only 63.3% often used electronic messages (via WhatsApp), 43.3% used an email account and 20.1% reported internet access at home.

**Table 1:** Self-reported participant characteristics

	**All participants** **(*n* = 1556)**	**Can you tell your BP levels?**		***p* value**
		Yes (*n* = 645)	No (*n* = 911)	
Mean age, years (sd)	66.5 (12.1)	65.7 (12.5)	67.2 (11.7)	0.014
Female sex, n (%)	1153 (74.4)	481 (74.7)	672 (74.3)	0.847
Mean body mass index, Kg/m^2^ (sd)	27.0 (5.5)	27.1 (5.4)	27.0 (5.8)	0.749
Low socio-economic strata^ab^, n (%)	791 (64.0)	315 (57.2)	476 (69.5)	< 0.001
Up to 5 years of school^b^, n (%)	780 (63.1)	318 (57.0)	462 (68.1)	< 0.001
Receiving care at public institutions, n (%)	1219 (78.3)	557 (86.4)	662 (72.7)	< 0.001
History of, n (%)				
Dyslipidemia	441 (28.3)	172 (26.7)	269 (29.5)	0.217
Diabetes mellitus	284 (18.3)	125 (19.4)	159 (17.5)	0.332
Chronic kidney disease	189 (12.2)	80 (12.4)	109 (12.0)	0.794
Acute coronary syndrome	125 (8.0)	48 (7.4)	77 (8.5)	0.470
COPD	106 (6.8)	37 (5.7)	69 (7.6)	0.156
Stroke	44 (2.8)	21 (3.3)	23 (2.5)	0.391
Access to, n (%)				
Cell phone	1503 (96.6)	637 (98.8)	866 (95.1)	< 0.001
WhatsApp messages	991 (63.7)	434 (67.3)	557 (61.1)	0.013
E-mail	667 (42.9)	258 (40.0)	409 (44.9)	0.055
Internet at home	323 (20.1)	174 (27.0)	149 (16.4)	< 0.001

*COPD* Chronic obstructive pulmonary disease

^a^320 participants with missing data for education level and socioeconomic strata (variables introduced later in the questionnaire)

^b^Levels 1–2 out of six established by the city for billing public utilities

As shown in Table 1, participants with knowledge of their BP levels were slightly younger, more often lived in higher socio-economic strata neighborhoods, had more than 5 years of education and were seen at public health care facilities. This group more often used information technologies, including cell phones, message exchange services via WhatsApp (but not email), and had home internet access.

**Fig. 2:**
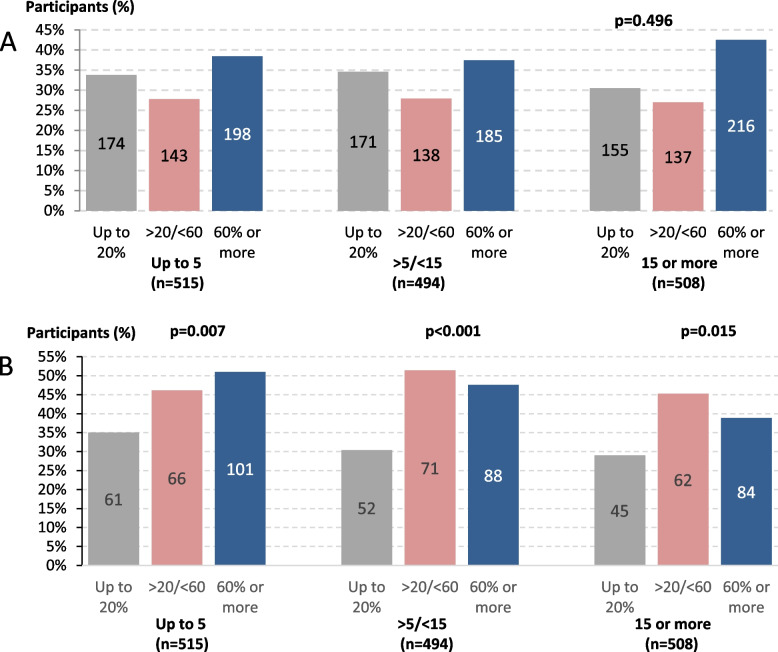
**A** Distribution of study participants by years after diagnosis (outer categories) within reported percentage of follow up visits done with the same doctor (inner categories). **B** Knowledge of blood pressure levels found by the same categories

Figure 2 (upper section A) shows the distribution of study participants by categories of time from being diagnosed (outer categories) and the frequency of visits done with the same doctor (inner categories). Among 1517 patients with both type of responses available, there were similar proportions (38.5%, 37.5% and 42.5%, *p* = 0.496) doing 60% of more of their visits with the same doctor across 3 categories of time from being diagnosed (up to 5 years, *n* = 515; between 5 and 15 years, *n* = 494, or more than 15 years, *n* = 508, respectively). The lower section (B) of the figure describes the association between reporting BP levels and the same categories. Those patients seen more often by the same doctor significantly reported higher knowledge of their BP levels. This observation was consistent for all categories of reported time with hypertension (*p* = 0.007 for those up to 5 years, *p* < 0.001 for those between 5 and 15 years, and *p* = 0.015 for those with more than 15 years of diagnosis, respectively)

Overall, 645 patients (41.4%, 95% confidence intervals 39.0 to 43.9) said they knew their BP levels. Reported values of systolic BP ranged from 92 to 190 mmHg (mean 130, sd 20.3), whereas values of diastolic BP ranged from 40 to 160 mmHg (mean 75, sd 20.1). Five (0.8%) of these participants were unable to report specific values, and three did not report the last time they checked their BP levels.

**Fig. 3:**
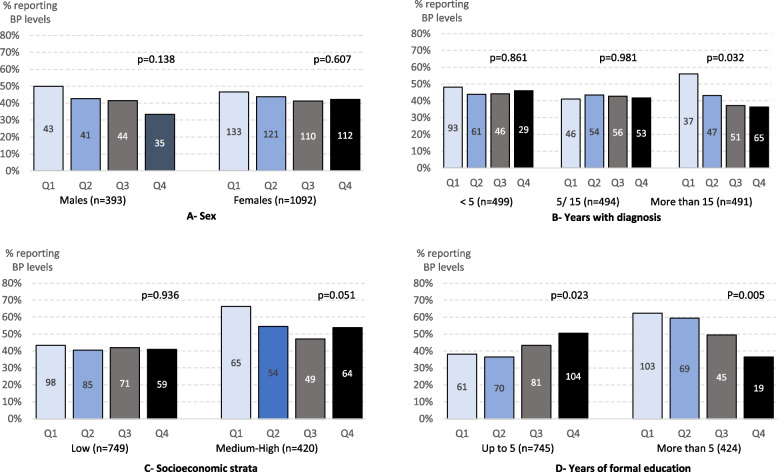
Variations in knowledge of PB levels across age quartiles (Q1: 22-59, Q2: 60-67, Q3: 68-74, Q4: 75-100 years) by (**A**) sex, (**B**) years of diagnosis, (**C**) socioeconomic strata, or (**D**) years of education

Age was inversely related to knowledge of BP levels. When exploring its variation by other sociodemographic factors, we observed that younger patients with more than 15 years of diagnosis, living in higher socioeconomic strata, or with or more years of education more often reported their BP levels. Figure 3 shows these variations.

**Fig. 4:**
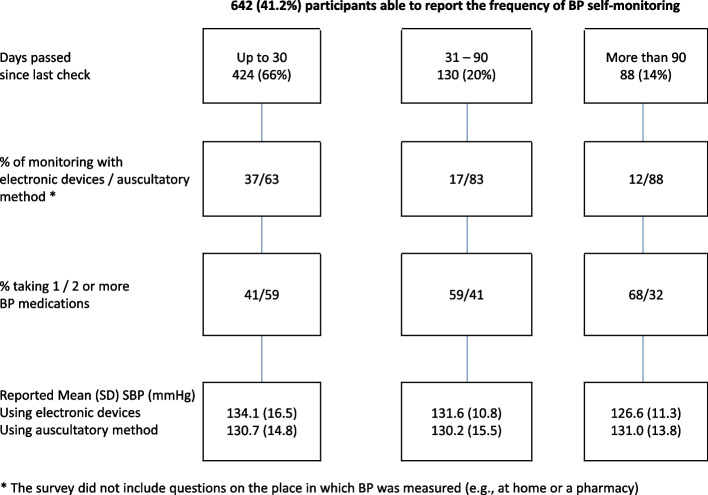
Main features associated with study participants reporting their BP levels

Figure 4 describes some key features among 642 participants able to report the frequency of their BP monitoring. Two-thirds had checked their BP levels within 30 days of the date of the interview, 20% say they had done it 30 to 90 days, and 14% more than 90 days ago. Use of BP electronic devices and having 2 or more medications prescribed were more often reported among those with more frequent monitoring. Also, reported systolic BP values tended to be higher (and statistically more disperse) among those doing more frequent monitoring with electronic devices.

Table 2 shows the answers to four questions on general understanding of hypertension among participants, classified using three different criteria: by years with the diagnosis, percentage of visits done with the same doctor, or knowledge of BP levels. Two-thirds of the patients answered that the word hypertension stood for having high BP, but only 8% could recognize that the two values used were for systolic and diastolic BP; 43% identified the BP treatment goals as below 140/90 mmHg, and 58% indicated that additional medication may be needed if BP was out of these goals. Overall, 26% correctly answered 3–4 of these questions. Importantly, while these proportions had little or no variation by years of diagnosis, they differed more sharply among patients who reported (or not) their knowledge of BP levels compared with the other forms of classification.

**Table 2:** General knowledge on hypertension among study participants using three different forms of classification

**Question (% of the overall population giving a right answer)**	**Forms of classifying participants** **(% with a right answer by category, Χ**^**2**^** statistic)**		
	**By years being diagnosed (3 incremental levels)**	**By % of follow up visits with the same doctor (3 incremental levels)**	**By knowledge of blood pressure levels (Yes/No answer)**
Hypertension stands for high blood pressure (67)	63/69/68 (6.1)*	57/74/70 (32.7)*	77/59 (55.7)**
The two BP numbers stands for systolic and diastolic BP (8)	8/7/7 (0.1)	4/9/9 (11.9)**	13/3 (52.3)**
BP treatment goals are < 140 and < 90 (43)	41/41/45 (2.1)	33/48/46 (27.1)*	56/32 (87.8)**
If BP is not within goals additional medications may be needed (58)	59/59/58 (0.3)	54/65/57 (10.4)*	67/52 (35.1)**
3–4 of the above questions right (26)	26/26/26 (0.1)	17/34/28 (36.8)**	40/15 (128.4)**

Knowledge classified by years after diagnosis (left), % of follow up visits done with the same doctor (center) or reporting knowledge of blood pressure levels (right)

^*^p < 0.05, **p < 0.001

**Fig. 5:**
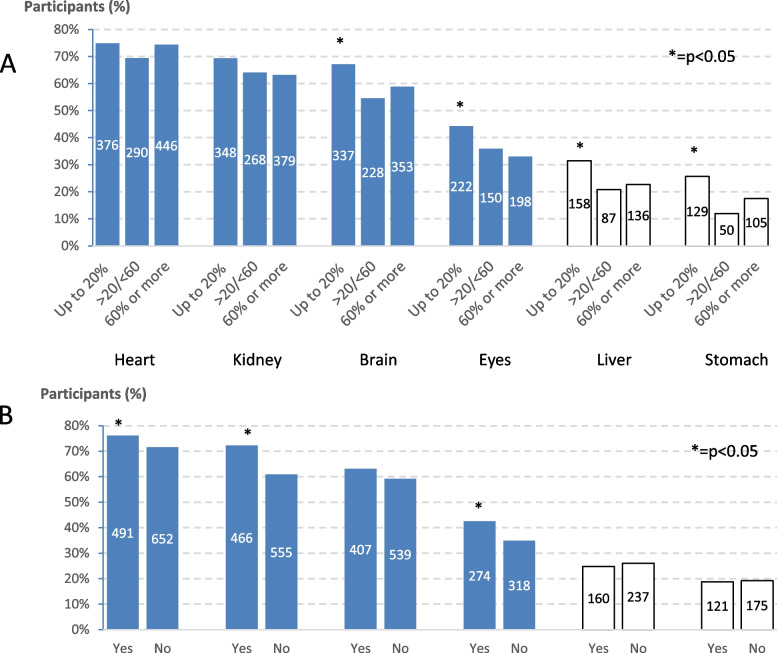
Report of organs potentially affected by hypertension when classifying participants by (**A**) percentage of follow up visits done with the same doctor or (**B**) by knowledge of blood pressure levels

Figure 5 shows the report of organs potentially affected by hypertension when classified by proportion of visits with the same doctor (upper section) or knowledge of BP levels (lower section). Participants correctly identified most BP target organs (more often the heart, and decreasingly kidney, brain and eyes). However, over 20% also said that hypertension could cause damage to the liver or stomach. The proportions of patients who identified correctly these organs tended to decrease by the frequency of visits with the same doctor, but to be higher among those reporting knowledge of BP levels.

## Discussion

This survey shows that, despite long-standing recommendations, still a minority (41%) of the participant hypertensive patients managed in Bogotá were able to report their BP levels. Those in that capacity were in general living in better conditions (i.e., higher socio-economic strata neighborhoods, had more education) and had more access to information technologies. Interestingly, more patients from public health care institutions reported their BP levels than those from private facilities (86.4 Vs. 72.7%), a finding potentially associated with other, unexplored factors that deserves further investigation.

Our data suggest that in this context of care, reporting BP levels may be a good, single marker of better care for hypertensive patients. Firstly, we observed a positive dose–response relationship between frequency of monitoring and higher use of BP electronic devices, receiving more often 2 or more medications and more between-patient variability (which suggest less rounding) of the BP levels reported. Secondly, significantly more patients gave right answers on general aspects of hypertension when classified by the ability to report their BP levels. Thirdly, identifying correctly the target organ damage was (despite a worrisome 25% proportion of false-negative answers) at least as good when classifying patients by reporting their BP levels or by the frequency of follow up visits with the same doctor. All the above aspects give validity to reporting BP levels as a variable to infer adequacy of care for hypertension in our communities. This may also be a potential target for future campaigns among hypertensive patients.

A concerning finding from this study was the lack of continuity in the care offered to many hypertensive patients in Bogotá. Only 40% of the study population reported frequent contact (at least 60% of the times) with the same health care provider. Unfortunately, this proportion did not change across categories of time with hypertension (two-thirds of our population had over 5 years knowing of their diagnosis). In contrast, reporting more often follow-up visits with the same doctor was positively associated with self-report of BP levels no matter the time of being diagnosed. A relationship between continuity of care and BP control has already been described among Colombian patients with hypertension [25]. This strongly suggests that, as expected, a more continuous follow up, namely having a family doctor, caring for hypertensive patients will improve knowledge of their condition, and potentially the clinical benefit from treatment.

Other investigators have approached hypertensive patients to assess their knowledge of the condition in general. There are questions in common among studies (some have developed their own instruments) that are concordant with what we asked. Estrada and cols found that 42% (of 980 patients interviewed in Spain) know their target systolic BP [26], similar to our results. Almas and cols in Pakistan reported that 46% (of 447) hospital-based patients understand that hypertension stands for high blood pressure (our figure was 67%, with a multiple-choice question) [27], but also in that study two-thirds of their patients knew the BP treatment goals (43% in our case). Likewise, these investigators asked for the target organs and reported that the heart and the kidney were more and less often mentioned (62.6 and 30.1% respectively, similar to our results), with the brain and the eyes in the middle position in that list. Amer and cols, also in Pakistan, reported that 24% of their 384 patients had good knowledge of the condition [28]. We found that 26% of our participants answered 3 or 4 out of four questions. Notably, Rahman in India found a consistent relationship between control and knowledge by exploring different aspects [29]. Those with more knowledge were in a better social position (more often government employees, with their health expenses covered). Despite differences in population and design with our study, most of these results show some similarity. However, self-report of BP levels had not been part of their questions.

### Strengths and limitations

Our study is, to the best of our knowledge, the first report of this kind of features of the day-to-day care of hypertensive patients in Colombia. It included a representative sample within both regimes of the health care system with a substantial number of outpatient clinics in the city of Bogotá. Our survey included mostly population of low socio-economic strata, usually managed at public facilities, receiving subsidized health care in Colombia. Although Bogotá covers a much smaller proportion of its population with subsidized health care (18% compared with 46% nationwide) [30], the features of the paid-for and subsidized health care settings throughout the country may be similar. Moreover, based on several study reports showing similar rates of awareness treatment and control of hypertension in LMICs, our results may extrapolate to the situation of hypertension in other nations of similar characteristics, either in South America, or even other continents.

Our study recorded self-reported information, directly from patients. While this may be a limitation for some responses (participants could have reported less those traits perceived as non-desirable such as low level of education, comorbidities, excess of body weight, among others), we see as strength exploring knowledge on hypertension directly from patients. Although in-person additional interviews were initially planned for other phases of this project, they were not feasible at the time, because of the emergence of Covid-19 pandemic. However, this analysis included several cross-validation features giving support to our inferences. We could not collect details of the calls with participants, such as dates, times, and durations. This information could have been useful to identify patterns in patient responses to improve the conduct of this or future telephone surveys. Finally, as we did not have in-person contact, we did not measure the “actual” BP levels of our participants. Adding the rates of BP control would have strengthened our inferences regarding knowledge BP levels as marker (in this case, of patients on treatment goals, but rather on knowledge of their condition).

### Implications

Decision makers should take our findings as a call to action in different aspects of care of patients with hypertension. Administrative efforts are needed to bring more continuous care and make more stable patient-physician relationships in Bogotá. This is not only important to promote knowledge of their conditions, but to improve communication, and thus adherence to recommendations. On the other hand, achieving more self BP monitoring will need communications beyond the contact in doctors’ offices. Actions to appoint doctors to groups of patients will be a step forward, but perhaps assembling hypertension clinics, or hypertension teams could make a higher impact. Finally, having patient education programs at a national level with patient information websites and leaflets, and possibly tax relief and incentives to purchase a home blood pressure monitor could be considered, at least starting with high-risk population.

Our results, only a first step in this field, underline the need for further research exploring knowledge on other recommendations. There is a need to understand the process of knowledge transfer among hypertensive patients within health care systems of countries like Colombia.

In conclusion, in our survey representing mostly public health care facilities in Bogotá, 41% of hypertensive patients, most of them with over 5 years of diagnosis, were able to report their own BP levels. Those patients were slightly younger, lived in higher socio-economic strata neighborhoods, had more formal education, and more access to information technologies. A more frequent self-monitoring was associated with using electronic devices and receiving two or more medications. Both self-report of BP levels and having more often contact with the same doctor were associated with a better knowledge of hypertension. Strategies for hypertensive patients to have more continuous contact with doctors, improve awareness of their BP levels and self-monitoring are warranted for a better knowledge of their condition.

### Supplementary Information

**Additional file 1.**

## Data Availability

The datasets used and analysed during the study are available from the corresponding author on reasonable request.

## Abbreviations

BP: Blood pressure
LMIC: Low- and middle-income countries
