# Supplementary material for: Reporting of blood pressure levels and self-monitoring practices: a survey among outpatients diagnosed with hypertension in Bogotá, Colombia
Source: BMC Prim Care. 2023 Sep 14;24:185. doi: 10.1186/s12875-023-02111-8 (PMC10503071; doi:10.1186/s12875-023-02111-8)
Supplement: Supplementary file 1 — Additional file 1. [file 12875_2023_2111_MOESM1_ESM.docx]

**Case Report Form - Knowledge on hypertension section ***

1. **Blood Pressure Levels**

Do you know your blood pressure (BP) levels? Yes ___ No ___

Can you tell your last blood pressure reading? Systolic _____ / Diastolic ____ mmHg

How long ago was that reading obtained? ____ Days ____ Months

Please describe the method was used to measure your BP

____ automatic device ____ auscultatory (stethoscope)

Where was it measured? ___ Physician’s office ___ Pharmacy ___ Home ___

1. **General questions on hypertension**
2. What is hypertension for you **(mark one)**

- High blood pressure
- Flushing of the face
- High stress
- High blood sugar
- I Don´t know

1. Regarding your blood pressure readings, what are the two numbers given to you **(tick all that apply)**

- The first number is systolic blood pressure
- The first number is diastolic blood pressure
- The second number is systolic blood pressure
- The second number is diastolic blood pressure
- I Don´t know

1. Which blood pressure levels are considered normal **(mark one for each row)**

1^st^ number ___ over 140 ___140 ___ below 140 ___ I Don´t know

2^nd^ number ___over 90 ___ 90 ____ below 90 ___ I Don´t know

1. If the blood pressure is too high, which organs do can be affected **tick all that apply)**

- Brain
- Heart
- Kidneys
- Eyes
- Liver
- Stomach
- I Don´t know

1. If your blood pressure remains out of control, you may need additional medication to control it

__ False __ True ___ I Don´t know

*English version of the questionnaire that research assistants applied to study participants
